# Engineered TALE Repeats for Enhanced Imaging‐Based Analysis of Cellular 5‐Methylcytosine

**DOI:** 10.1002/cbic.202000563

**Published:** 2020-11-06

**Authors:** Álvaro Muñoz‐López, Anne Jung, Benjamin Buchmuller, Jan Wolffgramm, Sara Maurer, Anna Witte, Daniel Summerer

**Affiliations:** ^1^ Faculty of Chemistry and Chemical Biology Dortmund University Otto-Hahn Strasse 6 44227 Dortmund Germany

**Keywords:** DNA methylation, epigenetics, imaging probes, membrane-less organelles

## Abstract

Transcription‐activator‐like effectors (TALEs) are repeat‐based, programmable DNA‐binding proteins that can be engineered to recognize sequences of canonical and epigenetically modified nucleobases. Fluorescent TALEs can be used for the imaging‐based analysis of cellular 5‐methylcytosine (5 mC) in repetitive DNA sequences. This is based on recording fluorescence ratios from cell co‐stains with two TALEs: an analytical TALE targeting the cytosine (C) position of interest through a C‐selective repeat that is blocked by 5 mC, and a control TALE targeting the position with a universal repeat that binds both C and 5 mC. To enhance this approach, we report herein the development of novel 5 mC‐selective repeats and their integration into TALEs that can replace universal TALEs in imaging‐based 5 mC analysis, resulting in a methylation‐dependent response of both TALEs. We screened a library of size‐reduced repeats and identified several 5 mC binders. Compared to the 5 mC‐binding repeat of natural TALEs and to the universal repeat, two repeats containing aromatic residues showed enhancement of 5 mC binding and selectivity in cellular transcription activation and electromobility shift assays, respectively. In co‐stains of cellular SATIII DNA with a corresponding C‐selective TALE, this selectivity results in a positive methylation response of the new TALE, offering perspectives for studying 5 mC functions in chromatin regulation by *in situ* imaging with increased dynamic range.

## Graphical abstract

**Two‐TALE test**: Engineered transcription‐activator‐like effector (TALE) repeats can be used to design programmable receptors for improved in situ imaging‐based analysis of 5 mC in user‐defined DNA sequences. Pairs of natural 5 mC‐discriminating and engineered 5 mC‐binding TALEs allow nucleotide resolution analysis of 5 mC with response of both TALEs. This provides an impulse for the design of programmable imaging probes for studying 5 mC functions in chromatin regulation with increased dynamic range.

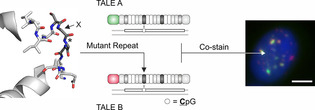

5‐Methylcytosine (5 mC, Figure 1a) is the main epigenetic modification of mammalian DNA and a central regulator of transcription, cell differentiation and development.^[1]^ Methylation is introduced by DNA methyltransferases (DNMT) mainly at CpG dinucleotides, and mis‐regulation of methylation is an early event in carcinogenesis.^[2]^ In addition to the main strategy for studying 5 mC functions, that is, its high‐resolution analysis and mapping in purified genomic DNA,^[3]^ methods for the imaging‐based *in situ* analysis of cellular 5 mC with high resolution have recently been reported. These promise to enable direct co‐observations of 5 mC and other imageable chromatin features at user‐defined genomic positions of single cells for *in situ* correlation studies.^[4]^

**Figure 1:**
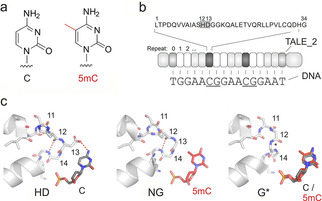
DNA recognition of TALEs. a) Chemical structures of cytosine and 5‐methylcytosine. b) Features of a TALE used in this study. An example repeat sequence is on top with the RVD in box. 5′‐T nucleotide is bound by the noncanonical repeat 0 and is thus not counted in target sequences. c) Crystal structures of DNA‐bound TALE RVDs HD and NG,^[9]^ and model of RVD G*^[10]^ bound to C or 5 mC, respectively.

Co‐stains of global 5 mC and of a target sequence are traditionally based on two separate generic probes such as antibodies and fluorescence *in situ* hybridization (FISH) probes.^[5]^ In contrast, approaches to combine 5 mC and target sequence selectivity in one probe scaffold have recently been reported, enabling selective analysis of 5 mC only at the target sequence with high resolution. For example, combination of programmable DNA‐binding proteins with methyl‐CpG‐binding domains in fluorescence complementation designs^[6]^ enabled live cell detection of 5 mC at user‐defined loci.^[7]^ Alternatively, FISH probes equipped with long chelator linkers for OsO_4_‐mediated crosslinking of 5 mC have been employed, though requiring harsh, oxidative crosslinking conditions.^[8]^

To enable imaging‐based *in situ* analysis of cellular 5 mC with nucleotide and strand resolution, we recently developed an approach based on transcription‐activator‐like effector (TALE) proteins^[11]^ serving as imaging receptors.^[12]^ TALEs bind one strand of a DNA duplex via a modular domain of repeats, each recognizing one nucleobase via a repeat variable di‐residue (RVD, Figure 1b).^[13]^ For example, cytosine is recognized by the RVD HD, whereas T (and 5 mC) are recognized by the RVD NG (Figure 1c). Repeats with selectivity for epigenetically modified nucleobases or with universal binding to any nucleobase have been developed,[^10^, ^14^] and employed for the analysis of cytosine modifications in purified genomic DNA.[^14e^, ^15^] Our imaging approach is based on two‐color co‐stainings of mammalian cells with fluorescent TALEs,^[16]^ one of which being labeled with GFP and binding to target CpG cytosines with HD RVDs that are blocked by 5 mC. The other TALE is labeled with mCherry and differs from the first TALE only by the replacement of the CpG‐interacting HD RVDs by the universal RVD G* (Figure 1c). This TALE serves as 5 mC‐unresponsive control to dissect differences in 5 mC at a target locus from differences in target DNA accessibility (e. g., by chromatin condensation) as a biological consequence of 5 mC differences.

We here report the development of enhanced 5 mC‐selective repeats by *in vitro* screening, and their integration into fluorescent TALEs. This enables an advancement of our imaging approach by replacing the universal TALE in the pair by a 5 mC‐selective TALE, resulting in a methylation response of both TALEs and thus a new impulse for *in situ* 5 mC studies with enhanced dynamic range by cellular imaging.

We constructed a library of “size‐reduced” TALE repeats containing an RVD deletion, since this general approach has recently been particularly successful for the discovery of novel nucleobase selectivities.[^10^, ^14d^, ^14e^] We deleted amino acid 13 and introduced a single random position at residue 12 that covered 11 amino acids with polar side chains. To allow a greater variety of polar interactions within the loop, we additionally introduced an S11N mutation. Figure 2a shows the library design in a repeat model of RVD G* (Figure 2b shows the repeat sequences).

**Figure 2:**
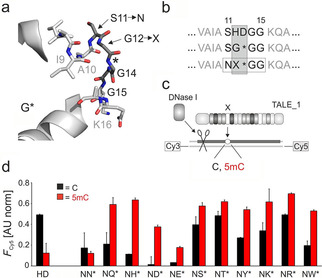
Library design and screening assay for the development of 5 mC‐selective TALE repeats. a) Positions targeted for deletion or randomization in a model of repeat G*;^[10]^ *: deletion; X: random position. b) Repeat sequences for RVDs HD, G*, and library. RVD positions in gray box. c) DNase I competition assay using Cy3/Cy5 doubly labeled DNA oligonucleotide duplexes with variable nucleobase (○) opposite a mutant repeat (X). d) Results of screening with DNase I assay conducted in duplicate with 0.5 μM of each TALE (RVDs indicated below), 0.1 μM DNA and 1 unit DNase I. The Cy5 fluorescence 25 min after DNase I addition is shown, background‐corrected by subtracting a control without TALE and normalized to a control w/o DNase I.

We assembled^[17]^ genes of “TALE_1” differing in a single mutant repeat, and encoding an N‐terminal GFP domain, an *AvrBs3*‐type N‐terminal region and a C‐terminal His_6_ tag for expression and Ni‐NTA purification in *Escherichia coli*.^[15]^ TALE_1 targets an 18 nt sequence in the zebrafish HEY2 gene (5′‐(T)CTTCCGTTTCCACATC‐3′) with the mutant repeat opposite the C of a single CpG dinucleotide.^[14a]^ We screened TALE_1 versions by a DNase I footprinting assay based on a Cy3/Cy5 dual‐labeled oligonucleotide duplex with a single C or 5 mC opposite the mutant TALE repeat (Figure 2c). In absence of bound TALE, DNase I catalyzes DNA cleavage resulting in decreased FRET from Cy3 to Cy5 as read‐out.^[18]^ A wild‐type (wt) TALE_1 positive control with RVD HD showed selective DNase I inhibition for the C‐duplex, but not the 5 mC duplex, confirming the selectivity of RVD HD (Figure 2d; Figure S1 in the Supporting Information shows full FRET kinetics). By contrast, the mutant repeats either showed no selectivity, or a variable degree of selectivity for 5 mC (Figure 2d). We previously made a related overall observation with a different repeat library containing a single amino acid deletion (using a different screening setup^[10]^), arguing for a model in that the deletion generally helps accommodating the 5‐methyl group of 5 mC (related to the small natural RVD NG and the single deletion RVD G*, compare to Figure 1c). The different residues at position 12 thereby modulate the size and surface structure of this pocket. Indeed, we observed different selectivities for the individual random site residues compared to our previous screen. For example, aromatic residues and acidic residues showed high selectivities, whereas hydroxyl‐bearing and basic residues rather showed no or lower selectivities (Figure 2d). Based on these and additional re‐screening experiments, we selected repeat ND* and the aromatic repeats NH*, NY* and NW* for further evaluation.

To study the behavior of the new TALE repeats in TALE_1 interacting with its target in the context of complex genomic chromatin, we conducted transcription activation assays in HEK 293T cells. We assembled mammalian expression plasmids with the respective TALE_1 genes fused to a VP64 transcriptional activation domain. We then co‐transfected them with a reporter plasmid with a cloned synthetic target sequence containing a single 5 mC at the target CpG as TALE_1 binding site (BS) directly upstream of a minCMV promoter controlling expression of a firefly luciferase gene (Figure 3a).^[19]^ Quantification of luciferase activity after 24 h cultivation showed no or low activity in absence of the BS, in absence of the TALE_1 plasmid, or in presence of a TALE_1‐VP64 construct with the correct RVD composition, but scrambled RVD order (scTALE_1, Figure 3b). By contrast, TALE_1 G* showed robust activation, similar to TALE 1 NG. Of the TALEs with novel repeats, ND* and NW* showed lower activation than the natural 5 mC‐binding NG RVD (Figure 3b). However, TALE_1 NH* and TALE_1 NY* showed higher activation, indicating increased binding to the 5 mC target DNA in the full complexity of HEK 293T cell nuclei, with relevance for cell staining applications.

**Figure 3:**
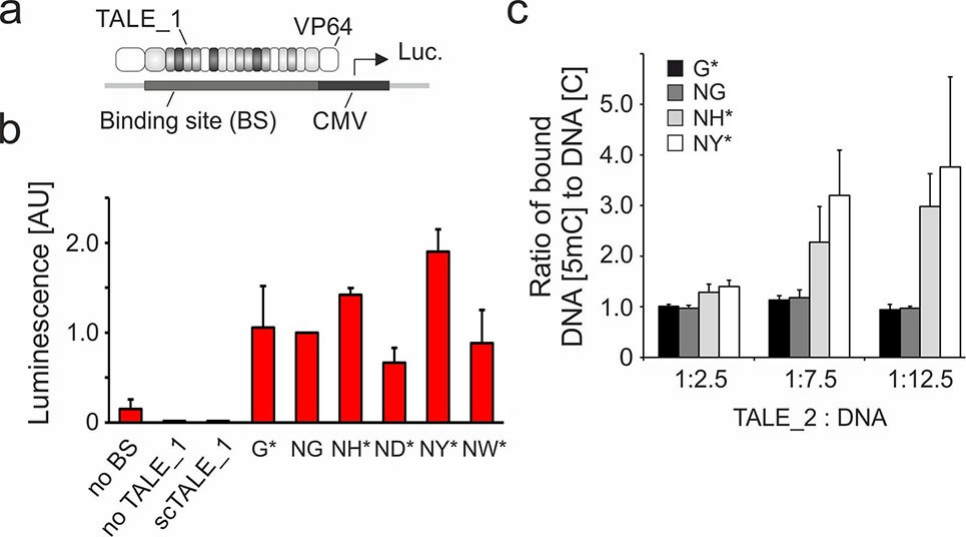
Characterization of engineered TALE repeats. a) Principle of 5 mC‐selective luciferase reporter assay based on a TALE_1‐VP64 fusion construct. b) Luminescence data from a transcriptional activation assay using Hey2‐targeting TALE_1 versions and luciferase reporter plasmid with methylated target sequence in HEK 293T cells. Error bars show the standard deviation of three independent biological replicates. c) 5 mC selectivity of SATIII‐targeting TALE_2 versions with standard and engineered repeats opposite two target CpGs in EMSA. Fraction of DNA‐bound TALE_2 versions in EMSA was quantified for different stoichiometries of TALE_2 s and target DNA duplex containing either 5 mC or C, and ratio between the two is depicted (bars show standard error of six independent experiments).

We further evaluated RVDs NH* and NY* in a sequence context usable for later cell staining and imaging‐based analysis of 5 mC. We chose to target a sequence from SATIII DNA, a class of clustered pericentromeric repeats that is the origin of nuclear stress bodies (nSB).^[20]^ These bodies are membrane‐less organelles that exhibit aberrant methylation in several cancers.^[21]^ We expressed in *E. coli* and purified versions of the previously evaluated SATIII‐binding TALE “TALE_2”, targeting the SATIII sequence 5′‐(T)GGAACGGAACGGAATG‐3′ with NH* and NY* repeats opposite the two CpG cytosines at positions 5 and 10. We then conducted electromobility shift assays (EMSA) with unlabeled DNA duplexes containing either C or 5 mC at the two target CpGs and with recording fluorescence of the GFP tag on the TALE protein. The new NH* and NY* TALE_2 versions showed pronounced 5 mC selectivity when DNA was in excess, whereas the standard G* and NG TALE_2 versions bound both C and 5 mC to a similar extent (Figures 3c and S2).

Having observed this improved 5 mC binding/selectivity of RVDs NH* and NY* in two different TALE‐contexts and *in vivo* and *in vitro*, we aimed to apply our novel repeats for imaging‐based 5 mC analysis of cellular SATIII DNA. The SATIII target sequence of TALE_2 can be targeted with high selectivity in a cellular context, indicated by co‐localization experiments with the SATIII marker protein HSF1 that is recruited to SATIII DNA upon a heat shock of 42 °C for 3 h (Figure 4a).^[20]^ We incorporated NH* or NY* repeats in place of G* repeats into our control TALE_2 that we previously used in our staining/imaging setup. In this setup, we employ pairs consisting of a GFP‐TALE_2 with HD repeats, and a control mCherry‐TALE_2 with universal G* repeats opposite the CpGs at positions 5 and 10 (Figure 4b). In co‐stains of fixed cell samples with differential SATIII methylation, an increased methylation is thereby indicated by a reduction of the HD TALE_2 fluorescence, whereas the fluorescence of the C/5 mC‐promiscuous G* TALE_2 serving as control remains unaltered.^[12]^ For generating cell samples with methylated and unmethylated SATIII, but minimal perturbation of the global methylation landscape, we employed our previous DNA‐methyltransferase construct “DNMT^act^” consisting of DNMT3a3 L^[22]^ fused to “TALE_0” targeting the SATIII sequence “(T)GATTCCATTCCATTCCATT” (differing from the target sequence of TALE_2 to avoid competition).^[12]^ Expression of DNMT^act^ in HEK 293T cells leads to an approximately sixfold increase in SATIII methylation compared to expression of a catalytically inactive E756A mutant^[23]^ (“DNMT^inact^”, Figure S4).^[12]^ We sorted DNMT^act^ or DNMT^inact^‐transfected HEK 293T cells for identical DNMT expression levels and then re‐plated, fixed and co‐stained them with equimolar amounts of HD GFP‐TALE_2, and either NY* or NH* mCherry TALE_2 (see Figure S3 for microscopy). We recorded signals for foci showing both mCherry and GFP fluorescence, and normalized for each TALE the signals to the mean fluorescence of the DNMT^inact^ foci. When compared to the HD versus G* TALE_2 co‐stain showing a significantly reduced HD TALE fluorescence for DNMT^act^ cells, we observed for the HD versus NY* co‐stain a similar trend, arguing for a selective response to increased 5 mC at the target CpGs (Figure 4c shows the histogram; data for the HD/G* co‐stain in Figure 4 are taken from ref. [12] and included for comparison. This co‐stain was conducted under identical conditions). By contrast, the NY* TALE showed similar fluorescence in both cell types, and thus rather behaved as universal TALE similar to TALE_2 G* (Figure 4d). In the HD versus NH* co‐stain, the HD TALE again showed a significant negative response to 5 mC. However, the NH* TALE showed a highly significant positive response with higher fluorescence for methylated cells (Figure 4d, see also box plots in Figure 4e). This establishes repeat NH* as 5 mC‐selective repeat that allows improved imaging‐based analysis of cellular 5 mC via TALE co‐stains with a double response of the employed TALEs.

**Figure 4:**
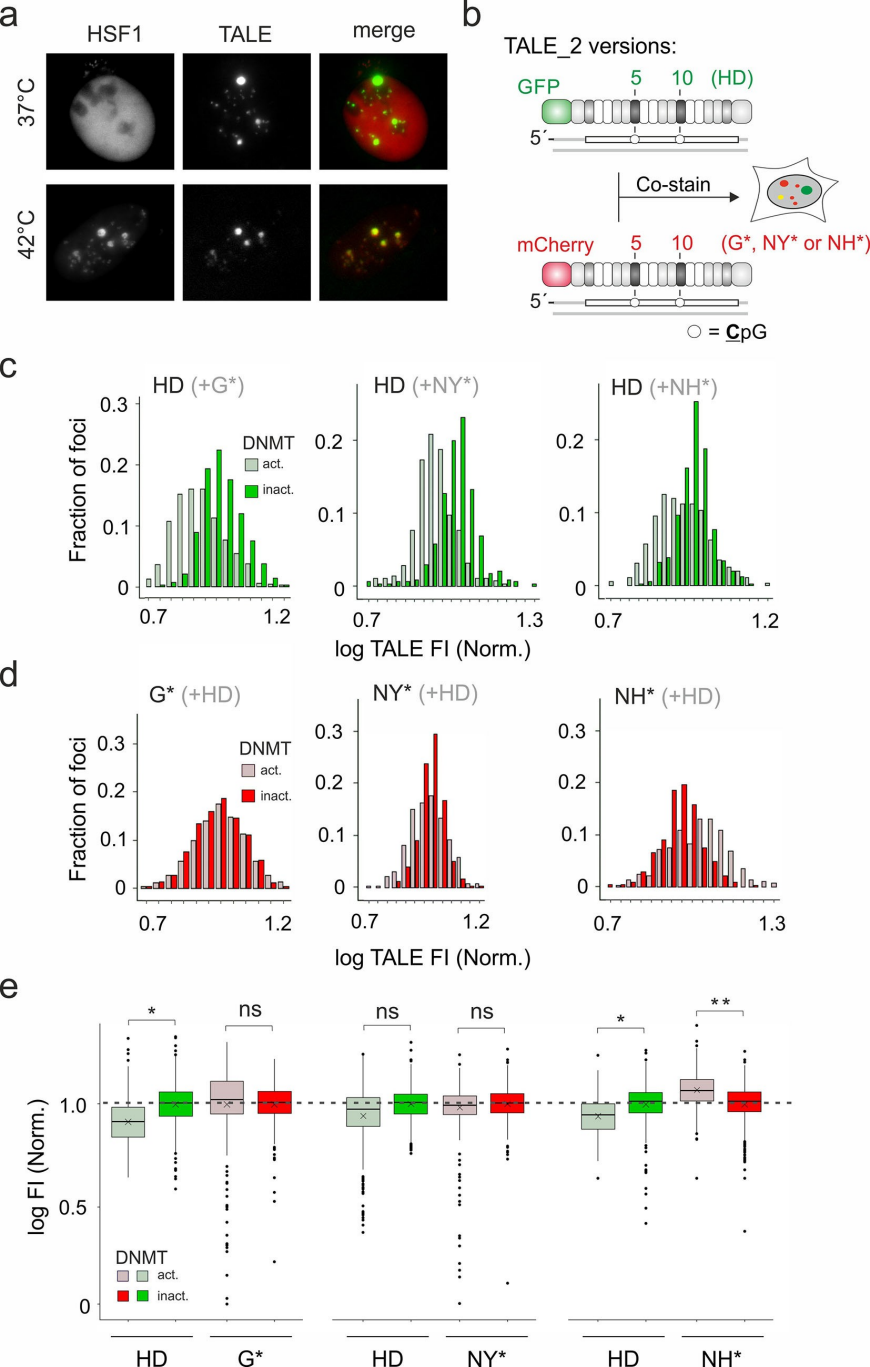
Evaluation of NY* and NH* repeats in 5 mC analysis at user‐defined CpG by cellular imaging. a) Imaging of cells expressing an mClover3‐TALE_2 version targeting CpGs with HD repeats and mCherry‐HSF1 with or without heat‐shock. b) Experimental setup and employed TALE_2 versions for co‐staining and imaging‐based 5 mC analysis. c) Histogram of HD TALE_2 FI of foci from DNMT^act^/DNMT^inact^ cells co‐stained with HD TALE_2 and (from left to right) TALE_2 G*, NY*, and NH*. For each TALE, log FI of each focus is normalized to the mean of log FI of all foci of DNMT^inact^ cells. d) Histograms of control TALE FI from co‐stains with HD TALE (corresponding to Figure 4d). Histograms in both Figure 4c and d are cropped for clarity and do not show outliers; these are fully shown in box plots of Figure 4e. e) Box plots of data from Figure 4c and d. Unpaired t‐test with * *p* <0.05; ns: not significant. *N*=5, 4 and 4 independent biological experiments with each ∼250 cells and >2000 foci.

In summary, we report engineered TALE repeats with reduced loop size for the selective recognition of 5 mC. A repeat with the sequence NH* in the repeat loop enables improved imaging‐based *in situ* analysis of 5 mC positions in user‐defined DNA sequences of single cells. We employ it in cellular co‐stainings with TALE pairs consisting of a TALE with C‐selective HD repeats and a TALE with 5 mC‐selective NH* repeats opposite the target C‐positions. This results in a positive response of the NH* TALE upon methylation of the target sequence, enabling its use as 5 mC‐reporter rather than mere control TALE. Our study thus provides a new impulse for the design of programmable receptors with selectivity beyond A, G, T and C for studying roles of DNA methylation in shaping chromatin functions *in situ*—with nucleotide, locus and cell resolution.

## Experimental Section

**TALE library generation**: Library of NX* repeat modules for repeat position 5 (pNX*5) was generated as previously described^[24]^ by restriction ligation using plasmid pHD5 as template. Briefly, pHD5 vector and respective annealed oligos (see the Supporting Information) were digested with NcoI and XhoI (New England Biolabs) and ligated with T4 DNA ligase. Repeat modules for position 10 (pNY*10 and pNH*10) were generated by Quikchange site‐directed mutagenesis (Agilent) with oligos o3541/o3542 and o3553/o3554 respectively, using pNN10 as template.^[12]^

**Vector construction**: The final entry vectors for Golden Gate Assembly pAni521, pÁlM1577, pÁlM1285, pÁlM1560, pcDNA3.1‐GoldenGate‐VP64 (Addgene, #47389) and pTALYM3 (Addgene, #47874) were generated previously.[^12^, ^16a^] The vectors pAni521 and pÁlM1577 are for expression in *E. coli* and purification of TALEs fused to eGFP or mCherry, respectively^2^. Vectors pÁlM1285 and pÁlM1560 are for mammalian expression of TALEs fused to DNMT3a3 L either wildtype or with E756 A inactivating mutation, respectively^2^. These two vectors contain an EBFP2 gene for sorting of transfected cells. Vector pTALYM3 was used for expression of TALEs fused to N‐terminal mClover3 in mammalian cells for live‐cell imaging and colocalization studies.^[16a]^ Vector pcDNA3.1‐GoldenGate‐VP64 allows mammalian expression of TALEs fused to VP64 transcriptional activator for targeted gene expression induction.^[25]^ Generally, TALEs were assembled into entry vectors by Golden Gate Assembly.^[17]^ Vector pHSF1‐mCherry for colocalization studies with HSF1 protein was constructed previously^2^. Generation of fLuc vectors with singly methylated TALE target sequence for transcription activation assays is described in section of luciferase assay.

**TALE expression and purification**: TALEs were expressed and purified as previously described.[^12^, ^26^] Briefly, BL21 DE3 Gold E. coli cells were transformed with expression plasmids, grown overnight in LB supplemented with carbenicillin (Carb) and diluted 50‐fold into the same medium. Cultures were induced with 0.5 mM IPTG at OD_600_ 0.8 and incubated overnight at 22 °C and 220 rpm. Cells were pelleted, resuspended in Deep Lysis Buffer (10 mM Tris⋅HCl, 300 mM NaCl, 2,5 mM MgCl_2_, 5 % DMSO, 0.2 % sodium lauroyl sarcosinate (AppliChem), 0.1 % Triton X‐100, pH 9) containing 1 mM PMSF, 1 mM of DTT and 50 μg/mL lysozyme (Sigma–Aldrich), and sonicated on ice (2×3 min 4 s on, 2 s off at 20 % amplitude). After centrifugation, supernatant extracted with HisPur™ Ni‐NTA Resin beads (ThermoFisher Scientific), washed with PBS, Lysis buffer+20 mM Imidazole+1 mM DTT, Lysis buffer+50 mM Imidazole+1 mM DTT, and eluted with Lysis buffer+500 mM Imidazole+1 mM DTT. Protein was dialyzed into TALE storage buffer (200 mM NaCl, 20 mM Tris, 10 % glycerol, pH 7.5)+1 mM DTT, aliquoted, snap‐frozen with liquid nitrogen and stored at −80 °C. Protein concentration was measured by a BCA assay (ThermoFisher Scientific).

**Library screening by DNaseI footprinting assay**: Assays were performed in 384‐well plate format (Greiner Bio‐one) as previously described.[^18^, ^24^] Briefly, HEY2 gene target sequences either methylated (o465) or unmethylated (o476) were hybridized with the reverse complementary oligo o1892 (5′‐Cy5‐ and 3′‐Cy3‐labeled) at a concentration of 200 nM in 3 μL Hybridization buffer (40 mM Tris⋅HCl (pH 8.0), 100 mM NaCl, 10 mM MgCl2, 0.2 mg/mL BSA, 10 % glycerol) by incubation at 95 °C for 5 min and then at room temperature for 30 min. TALE proteins were added in 3 μL TALE Storage Buffer (200 mM NaCl, 20 mM Tris⋅HCl (pH 7.5), 1 mM DTT, 10 % glycerol) to result in 0.5 μM final concentration and were incubated 30 min at room temperature. 6 μL of a mixture of 1 U DNase I (New England Biolabs) in DNase I buffer (20 mM Tris⋅HCl (pH 7.5), 5 mM MgCl_2_ and 0.2 mM CaCl_2_) were added per well and the plate containing the mix was placed immediately into a TECAN M1000 plate reader, pre‐heated at 37 °C. Excitation of Cy3 was performed at 552 nm and Cy5 Emission was acquired at 665 nm every 5 min over 1 h. Background Cy5 fluorescence was subtracted from control wells lacking TALE, and the ratio of Cy5 fluorescence of the TALE samples to that of a control without DNase I were plotted as relative Cy5 fluorescence.

**Luciferase assay**: Luciferase reporter plasmids were generated as previously described .^[19]^ Oligonucleotides o2520/o2501 containing the TALE target sequence methylated at the single CpG were hybridized at 10 μM each in 150 mM NaCl by heating at 95 °C for 5 min and cooling down at an interval of 3 °C/min to 10 °C. Hybridized oligos and vector pAnW755 (see the Supporting Information) were digested with SalI and SpeI (New England Biolabs) and purified using a PCR purification kit (Thermo Fisher Scientific). Digested inserts and vector were ligated with T4 ligase (New England Biolabs,) at 3 : 1 insert/vector ratio for 4 h at 16 °C. Products were directly used for transfection of HEK 293T cells. 1.4×10^4^ cells HEK 293T cells were seeded in a 96‐well plate (Merck) one day before transfection. Co‐transfection was performed with 100 ng of luciferase reporter plasmid and 100 ng of the respective TALE‐VP64 plasmid using Lipofectamine 2000 transfection reagent (ThermoFisher Scientific) with a reagent/DNA ratio of 3 : 1, following the manufacturer's protocol. For each condition, three replicates were prepared. Cells were lysed 24 h post transfection with lysis buffer containing 100 mM NaH_2_PO_4_ and 0.2 % Triton X‐100. After incubation on ice for 20 min, 20 μL of lysate were mixed with 90 μL of premixed Bright‐Glo^TM^ luciferase reagent (Promega) in a second 96 well plate. The luminescence of each well was analyzed by a Tecan Infinite M1000 plate reader (wavelength 380–600 nM). Luminescence data for different TALEs were normalized to the reaction of TALE NG.

**Electromobility shift assays**: EMSAs were performed as previously reported.[^12^, ^19^] A methylated (o3545) or an unmethylated (o3552) oligo with the target sequence of SatIII TALE as forward strand was hybridized to a complementary unmethylated reverse oligo (o3547) in Annealing Buffer (20 mM Tris, 50 mM NaCl, 5 mM MgCl_2_ and 5 % v/v glycerol, pH 8) by incubating 5 min at 95 °C, followed by a cool‐down to room temperature for 3 h. Annealed oligos at 15 to 400 nm concentration were incubated with 200 nM of the respective m‐Cherry‐TALE in TALE Binding Buffer (20 mM Tris⋅HCl pH 8, 50 mM NaCl, 5 mM MgCl_2_, 50 ng/μL salmon sperm DNA, 0.1 mg/mL BSA and 10 % glycerol) in 10 μL final volume. This mixture was incubated 1 h at room temperature and 30 minutes at 4 °C in the dark. Samples were run on pre‐run native polyacrylamide gels (0.5×TAE buffer, 8 % Rotiphorese gel 40 (Carl‐Roth), 0.1 % APS and 0.01 % TEMED) for 90 minutes at 4 °C in a Mini Protean vertical electrophoresis cell (Bio‐Rad) with a voltage of 70 V. mCherry fluorescence was recorded with a Typhoon FLA‐9500 laser scanner (GE Healthcare) using a 532 nm laser and LPG filter. Images were analyzed using ImageQuant TL 8.1 software (GE Healthcare).

**Cell culture and transfection**: HEK 293T and U2OS cells (Sigma Aldrich) were cultured at 37 °C under 5 % CO_2_ in DMEM (PanBiotech) supplemented with 10 % FBS (PanBiotech), 1 % of l‐glutamine 200 mM (PanBiotech) and 1 % of Pen/Strep (PanBiotech). For site‐directed methylation, HEK 293T cells were transfected with 10 μg of DNMT^act^ or DNMT^inact^ plasmid using FuGene 6 transfection reagent (Promega) following the manufacturer's protocol with a reagent/DNA ratio of 3 : 1. For live‐imaging and co‐localization studies after heat‐shock, 300 000 U2OS cells were seeded on a μ‐Dish 35 mm (ibidi, 81156) and transfected with 1 μg of TALE_0 assembled in pTALYM3 vector and 500 ng of pHSF1‐mCherry as described under section of luciferase assay.

**Flow cytometry**: Cells were incubated 48 h and trypsinized with Trypsin 0.05 % / EDTA 0.02 % (PanBiotech) for 5 min at 37 °C. After blocking with full DMEM medium, cells were centrifuged at 300 *g* for 10 min the supernatant was discarded and the cell pellet resuspended in 500–1000 μL of DPBS and transferred to a 5 ml FACS tube through a cell strainer (Falcon Corning, 352235). Sorting was performed with a Sony Cell Sorter model LE‐SH800SFP in targeted mode using the 405 nm laser (filter FL1 450/50, Optical Filter Pattern 2) for detection of EBFP2 from DNMT^act^ or DNMT^inact^ plasmids. Similar expression levels of EBFP2 on both samples were selected by proper gating. EBFP2+ cells were collected in tubes with Full DMEM+2 % of Pen/Strep (PanBiotech), and 100 000 cells/well were seeded in μ‐Plate 96 Well Black ibiTreat plates (ibidi) previously coated with 0.02 % of poly‐l‐lysine (Sigma‐Aldrich).

**TALE staining**: Cells seeded after sorting on a μ‐Plate 96 Well Black ibiTreat plate (ibidi) were incubated overnight prior fixation. Firstly, they were washed once with DPBS (PanBiotech) and then fixed with ice‐cold methanol at −20 °C for 10 min. After washing for 5 min with DPBS, cells were treated with 2 N HCl for 5 min at room temperature, followed by two washes with DPBS and incubation overnight with blocking buffer (DPBS‐T+1 % BSA). Samples were then stained with 200 μL of 0.8 nM of each (eGFP and mCherry) purified TALEs in DPBS+50 mM NaCl at room temperature for 30 min in the dark. After incubation, cells were washed four times with the same buffer for 5 min at room temperature and shaking at 300 rpm. Nucleus staining was performed by incubating the samples with 1 uL per well of Vectashield with DAPI in 200 μl of DPBS (Vector Laboratories, H‐1200) for 10 minutes at room temperature. Finally, cells were washed twice with DPBS for 5 min at room temperature and kept in DPBS for microscopy.

**Microscopy**: Microscopy was performed with an Olympus IX81 microscope coupled with a Hamamatsu model C10600‐10B‐H camera. Pictures were taken as z‐stack images of 6 μm (step size=0.3 μm) using a 60× oil objective covered with ibidi immersion oil (ibidi). Excitation settings for each channel were as follows: DAPI was acquired using the DAPI excitation filter (387/11), EGFP with excitation filter GFPFret (470/22) and mCherry with Cy3(560/25). The three fluorescence channels were detected using triple band dichroic DaFICy3 cube and the DaFICy3 quad band emission filter. Exposure times were 30, 100 and 50 ms for DAPI, EGFP and mCherry, respectively. For live‐cell imaging of co‐transfections with mClover3‐TALE and HSF1‐mCherry, plates were either incubated at 37 °C (control) or at 42 °C (heat‐shock) for 3 h before microscopy. Then, cells were immediately imaged as described above. Microscope chamber was pre‐warmed at the appropriate temperature with a heat unit.

**Image processing and analysis**: Foci detection and their respective intensity, area and subcellular location was analyzed from maximal intensity Z‐projections of image stacks (1344×1024 pixels, 12 bits) using the Fiji distribution of ImageJ.^[27]^ An out‐of‐interest region of each set of pictures was selected for subtraction of the mean background intensity in each channel of the stack. Nuclear regions were selected from DAPI channel (10 μm^2^ minimum area, circularity between 0.5 and 1.0). “GaussFit OnSpot” pluginn^[28]^ was used to analyze the intensity and size of the spots recorded in the GFP/FITC channel using elliptical shape and Levenberg Marquard fit mode with a 10 pixel rectangle half size. Signals outside the nucleus, spots with a prominence smaller than 30 (signal‐to‐noise ratio) or larger than 25 pixels were excluded from the analysis. Regions of Interest (Masks) generated by foci detection in GFP/FITC channel were directly applied to the mCherry channel to quantify the mean fluorescence intensity. For each nucleus, the number, size and intensity of the associated foci was recorded. All images were processed in batch using an ImageJ macro script that we created for a previous publication.^[12]^

**Data analysis and statistics**: The data was analyzed and plotted using R^[29]^ as previously described.^[12]^ Briefly, unsplit nuclei were excluded from the analysis by filtering out DAPI areas larger than 25 000 square pixels. For each TALE, mean fluorescence intensities of each focus were log transformed and then normalized to the average fluorescence intensity of all foci of the DNMT^inact^ sample of each independent experiment. Single cell data analysis was performed by calculating the average mean fluorescence intensity of all foci within a single nucleus and then log transformed and normalized as explained above. Graphs were plotted and statistical t‐test analysis were performed using ggplot2^[30]^ and ggpubr^[31]^ libraries, respectively. Additional t‐test analyses were carried out with GraphPad considering the number of independent experiments as sample size (*N*≥4 independent experiments in each case).

## Conflict of interest

The authors declare no conflict of interest.

## Supporting information

As a service to our authors and readers, this journal provides supporting information supplied by the authors. Such materials are peer reviewed and may be re‐organized for online delivery, but are not copy‐edited or typeset. Technical support issues arising from supporting information (other than missing files) should be addressed to the authors.

Supplementary
