# Supplementary material for: Engineered TALE Repeats for Enhanced Imaging‐Based Analysis of Cellular 5‐Methylcytosine
Source: Chembiochem. 2020 Nov 6;22(4):645–51. doi: 10.1002/cbic.202000563 (PMC7894354; doi:10.1002/cbic.202000563)
Supplement: Supplementary file 1 — Supplementary [file CBIC-22-645-s001.pdf]

ChemBioChem

Supporting Information

Engineered TALE Repeats for Enhanced Imaging-Based Analysis of Cellular 5-Methylcytosine

Álvaro Muñoz-López, Anne Jung, Benjamin Buchmuller, Jan Wolffgramm, Sara Maurer, Anna Witte, and Daniel Summerer*

Supporting Information

Table of Contents

Supplementary Tables S3

Supplementary Figures S6

Appendix 1 – Vector Maps S10

Supplementary Tables

Oligo Tables

Library construction of repeat module for position 5 by restriction-ligation of mutated annealed oligos.

Oligo ID	Description	Sequence (5' → 3')
o1691	VAIA**NN***GG	TTTTCCATGGCCTGACCCCGGACCAAGTGGTGGCTATCGCCAACAATGGC GGCAAGCAAGCGCTCGAAACGGTGCAGGAG
o1692	VAIA**NQ***GG	TTTTCCATGGCCTGACCCCGGACCAAGTGGTGGCTATCGCCAACCAGGGC GGCAAGCAAGCGCTCGAAACGGTGCAGGAG
o1693	VAIA**NH***GG	TTTTCCATGGCCTGACCCCGGACCAAGTGGTGGCTATCGCCAACCATGGC GGCAAGCAAGCGCTCGAAACGGTGCAGGAG
o1694	VAIA**ND***GG	TTTTCCATGGCCTGACCCCGGACCAAGTGGTGGCTATCGCCAACGATGGC GGCAAGCAAGCGCTCGAAACGGTGCAGGAG
o1695	VAIA**NE***GG	TTTTCCATGGCCTGACCCCGGACCAAGTGGTGGCTATCGCCAACGAAGGC GGCAAGCAAGCGCTCGAAACGGTGCAGGAG
o1696	VAIA**NS***GG	TTTTCCATGGCCTGACCCCGGACCAAGTGGTGGCTATCGCCAACAGCGGC GGCAAGCAAGCGCTCGAAACGGTGCAGGAG
o1697	VAIA**NT***GG	TTTTCCATGGCCTGACCCCGGACCAAGTGGTGGCTATCGCCAACACCGGC GGCAAGCAAGCGCTCGAAACGGTGCAGGAG
o1698	VAIA**NY***GG	TTTTCCATGGCCTGACCCCGGACCAAGTGGTGGCTATCGCCAACTATGGC GGCAAGCAAGCGCTCGAAACGGTGCAGGAG
o1699	VAIA**NK***GG	TTTTCCATGGCCTGACCCCGGACCAAGTGGTGGCTATCGCCAACAAAGGC GGCAAGCAAGCGCTCGAAACGGTGCAGGAG
o1700	VAIA**NR***GG	TTTTCCATGGCCTGACCCCGGACCAAGTGGTGGCTATCGCCAACCGTGGC GGCAAGCAAGCGCTCGAAACGGTGCAGGAG
o1701	VAIA**NW***G G	TTTTCCATGGCCTGACCCCGGACCAAGTGGTGGCTATCGCCAACTGGGGC GGCAAGCAAGCGCTCGAAACGGTGCAGGAG
o1746	Reverse primer	TTTTCTCGAGGGTCTCCTGCACCGTTTCGAGCGCTTG

Construction of modules pNY*10 and pNH*10 by Quikchange

Oligo ID	Description	Sequence (5' → 3')
o3541 fw	VAIA**NY***GG	GCTATCGCCAACTATGGCGG
o3542 rv	VAIA**NY***GG	CCGCCATAGTTGGCGATAGC
o3553 fw	VAIA**NH***GG	CCGCCATGGTTGGCGATAGC
o3554 rv	VAIA**NH***GG	GCTATCGCCAACCATGGCGG

DNaseI Footprinting Assay target sequence oligos

Oligo ID	Description	Sequence (5' → 3')
o476	Unmethylated	TGGATTCCCACTCTTCAGCCCCAGCGTTACAGCATCTTCAGTGGCTTCTT CCACCGTGAGCTCTTC**C**GTTTCCACATCC
o465	Methylated	TGGATTCCCACTCTTCAGCCCCAGCGTTACAGCATCTTCAGTGGCTTCTT CCACCGTGAGCTCTTC**N**GTTTCCACATCC **N**= 5mC

o1892	Reverse oligo	XGGATGTGGAAA**C**GGAAGAY	**X**= Cy5, **Y**= Cy3

Luciferase Assay target sequence oligos

Oligo ID	Description	Sequence (5' → 3')
o1899	Unmethylated	TTTTGTCGACTCTTC**C**GTTTCCACATCTACTAGTTTTT
o1900	Unmethylated	AAAAACTAGTAGATGTGGAAA**C**GGAAGAGTCGACAAAA
o2520	Mehtylated	TTTTGTCGACTCTTC**N**GTTTCCACATCTACTAGTTTTT **N**= 5mC
o2501	Mehtylated	AAAAACTAGTAGATGTGGAAA**N**GGAAGAGTCGACAAAA **N**= 5mC

EMSA target sequence oligos

Oligo ID	Description	Sequence (5' → 3')
o3552	Unmethylated	CCAATGGAA**C**GGAA**C**GGAATGGAATG
o3545	Methylated	CCAATGGAA**N**GGAA**N**GGAATGGAATG **N**= 5mC
o3547	Reverse	CATTCCATTCCGTTCCGTTCCATTGG

Sample sizes and number of experiments of staining experiments

Sample Size			
Co-staining	N (# of experiments)	# of cells	# of foci
HD vs G*	5	751	2560
HD vs NY*	4	725	3738
HD vs NH*	4	739	4086

Statistical data. Significance and p-values for unpaired t-test

HD + G* (N=5)	
	p-value
HD	* 0.0110
G*	ns 0.8907

HD + NY* (N=4)	
	p-value
HD	ns 0.2331
NY*	ns 0.3561

HD + NH* (N=4)	
	p-value
HD	* 0.0486
NH*	** 0.0036

TALE assemblies

TALE_1 (for DNase Footprinting Assay, assembled in pAni521)

	1	2	3	4	5	6	7	8	9	10	11	12	13	14	15	16	LR
HD	HD	NG	NG	HD	HD	NN	NG	NG	NG	HD	HD	NI	HD	NI	NG	HD	NG
NN*	HD	NG	NG	HD	NN*	NN	NG	NG	NG	HD	HD	NI	HD	NI	NG	HD	NG
NQ*	HD	NG	NG	HD	NQ*	NN	NG	NG	NG	HD	HD	NI	HD	NI	NG	HD	NG
NH*	HD	NG	NG	HD	NH*	NN	NG	NG	NG	HD	HD	NI	HD	NI	NG	HD	NG
ND*	HD	NG	NG	HD	ND*	NN	NG	NG	NG	HD	HD	NI	HD	NI	NG	HD	NG
NE*	HD	NG	NG	HD	NE*	NN	NG	NG	NG	HD	HD	NI	HD	NI	NG	HD	NG
NS*	HD	NG	NG	HD	NS*	NN	NG	NG	NG	HD	HD	NI	HD	NI	NG	HD	NG
NT*	HD	NG	NG	HD	NT*	NN	NG	NG	NG	HD	HD	NI	HD	NI	NG	HD	NG
NY*	HD	NG	NG	HD	NY*	NN	NG	NG	NG	HD	HD	NI	HD	NI	NG	HD	NG
NK*	HD	NG	NG	HD	NK*	NN	NG	NG	NG	HD	HD	NI	HD	NI	NG	HD	NG
NR*	HD	NG	NG	HD	NR*	NN	NG	NG	NG	HD	HD	NI	HD	NI	NG	HD	NG
NW*	HD	NG	NG	HD	NW*	NN	NG	NG	NG	HD	HD	NI	HD	NI	NG	HD	NG

TALE_1 (for Luciferase Assay, assembled in pcDNA3.1-GoldenGate-VP64)

	1	2	3	4	5	6	7	8	9	10	11	12	13	14	15	16	LR
G*	HD	NG	NG	HD	G*	NN	NG	NG	NG	HD	HD	NI	HD	NI	NG	HD	NG
NG	HD	NG	NG	HD	NG	NN	NG	NG	NG	HD	HD	NI	HD	NI	NG	HD	NG
NH*	HD	NG	NG	HD	NH*	NN	NG	NG	NG	HD	HD	NI	HD	NI	NG	HD	NG
ND*	HD	NG	NG	HD	ND*	NN	NG	NG	NG	HD	HD	NI	HD	NI	NG	HD	NG
NY*	HD	NG	NG	HD	NY*	NN	NG	NG	NG	HD	HD	NI	HD	NI	NG	HD	NG
NW*	HD	NG	NG	HD	NW*	NN	NG	NG	NG	HD	HD	NI	HD	NI	NG	HD	NG

TALE_2 (for imaging analysis, assembled in pAni521 or pÁlM1577)

	1	2	3	4	5	6	7	8	9	10	11	12	13	14	15	16	LR
HD/HD	NN	NN	NI	NI	HD	NN	NN	NI	NI	HD	NN	NN	NI	NI	NG	NN	NN
G*/G*	NN	NN	NI	NI	G*	NN	NN	NI	NI	G*	NN	NN	NI	NI	NG	NN	NN
NY*/NY*	NN	NN	NI	NI	NY*	NN	NN	NI	NI	NY*	NN	NN	NI	NI	NG	NN	NN
NH*/NH*	NN	NN	NI	NI	NH*	NN	NN	NI	NI	NH*	NN	NN	NI	NI	NG	NN	NN
HD/NG	NN	NN	NI	NI	HD	NN	NN	NI	NI	NG	NN	NN	NI	NI	NG	NN	NN

TALE_0 (For site-directed methylation, assembled in pÁlM1285 and pÁlM1560)

	1	2	3	4	5	6	7	8	9	10	11	12	13	14	15	16	17	18	LR
HD	NN	NI	NG	NG	HD	HD	NI	NG	NG	HD	HD	NI	NG	NG	HD	HD	NI	NG	NG

Supplementary Figures

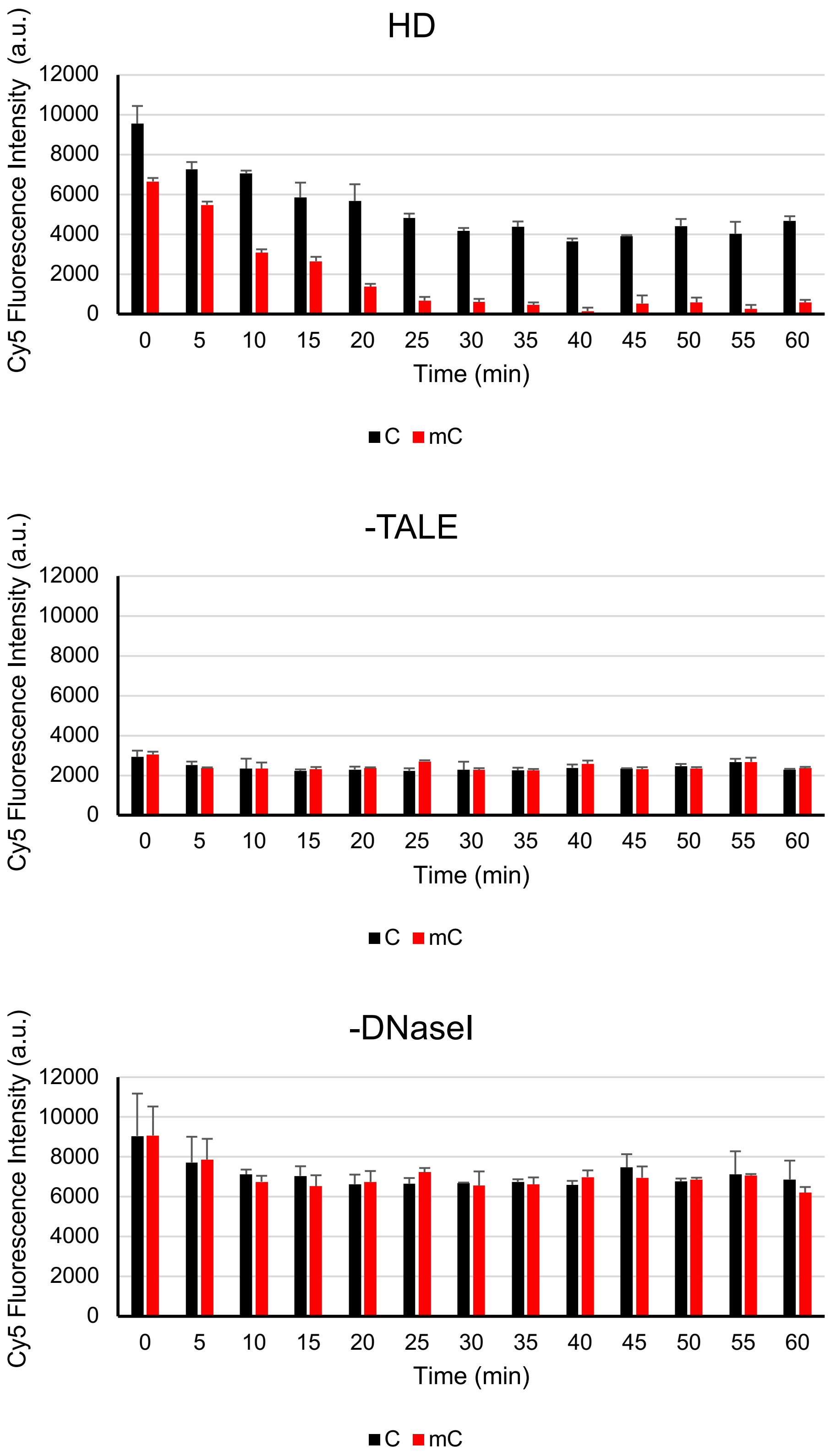

Figure S1. Fret Kinetics for TALE_1 HD and controls. Controls shown are performed under the same experimental conditions as TALE_1 HD either without TALE (middle) or without addition of DNaseI (bottom). Error bars show standard deviation.

Figure S2. EMSA

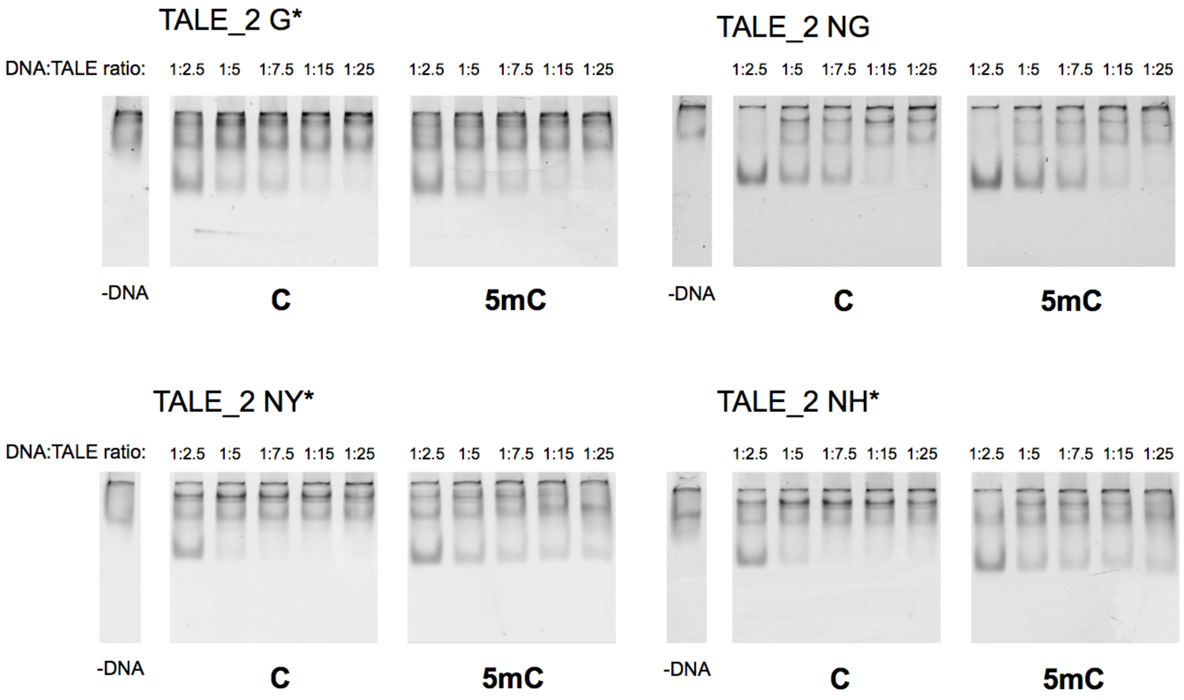

Figure S2. EMSA assays. EMSA with indicated, SATIII-targeting TALE versions bearing an N-terminal GFP and unlabeled DNA with C or 5mC opposite the target CpGs. Note that fluorescence of GFP is recorded.

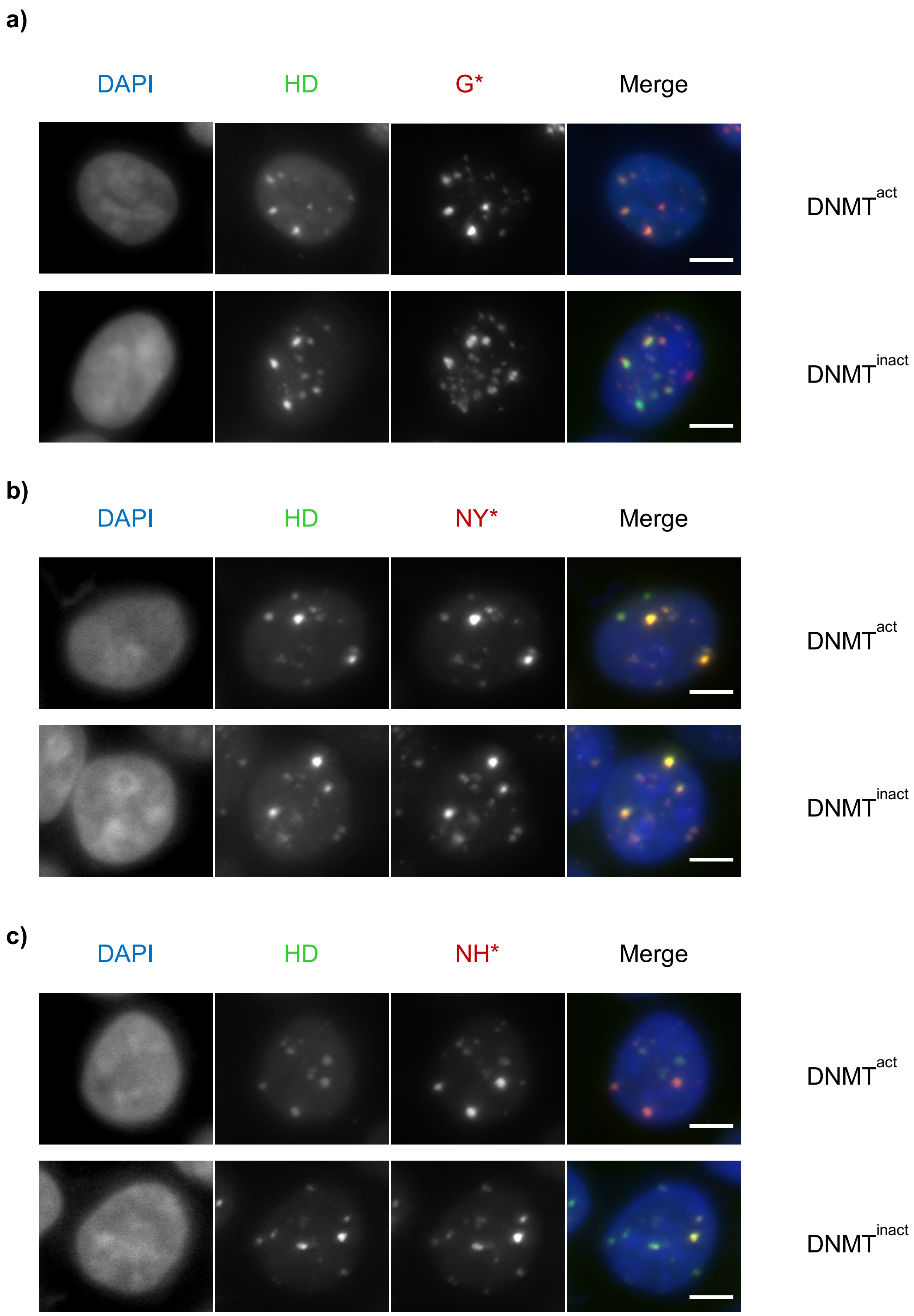

Figure S3. TALE staining. a) HEK 293T cells co-stained with TALE_2 bearing indicated repeats at positions 5 and 10 (HD in eGFP and G* in mCherry). **b)** Same as in a, but using NY* at positions 5 and 10 in the mCherry-TALE. **c)** Same as in a, but with RVD NH* for positions 5 and 10 of mCherry-TALE. Scale bar is 5 μm.

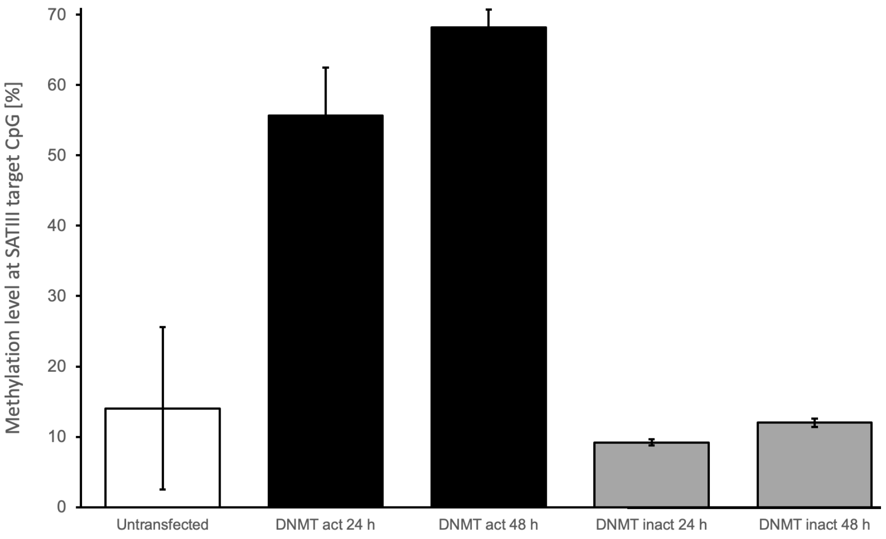

Figure S4. Methylation of SATIII target CpG. HEK293T cells were not transfected or transfected with the TALE_0-DNMT3a3L fusion protein “DNMT^{act}“ or the catalytically inactive version “DNMT^{inact}”. Shown are 5mC levels from bisulfite PCR and Pyrosequencing analysis of the SATIII target CpG at the indicated timepoints after transfection (Error bars from biological duplicates).

Appendix 1 – Entry Vector Maps

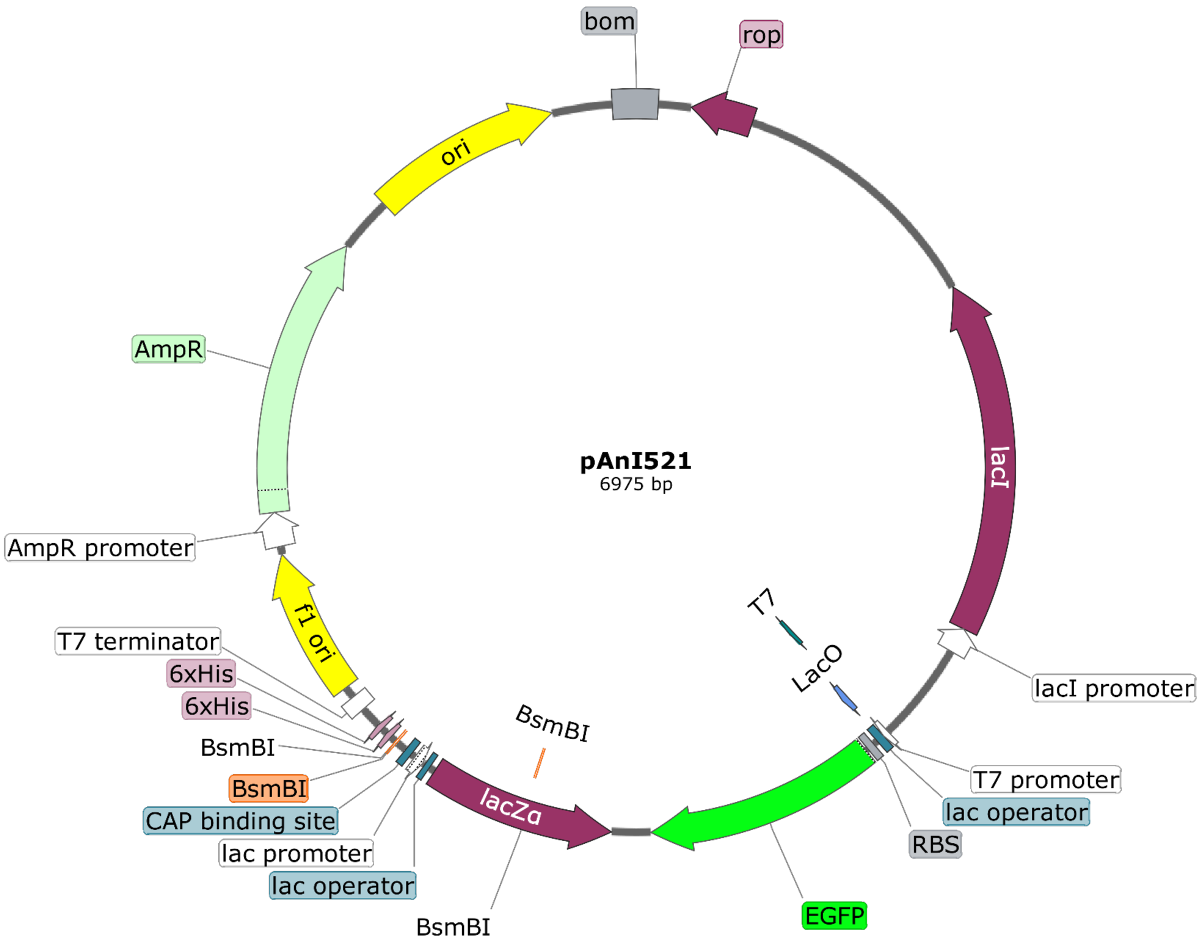

pAnI521

GG2 Entry Vector for bacterial expression of TALEs fused to N-terminal EGFP and C-terminal 6xHis for protein purification.

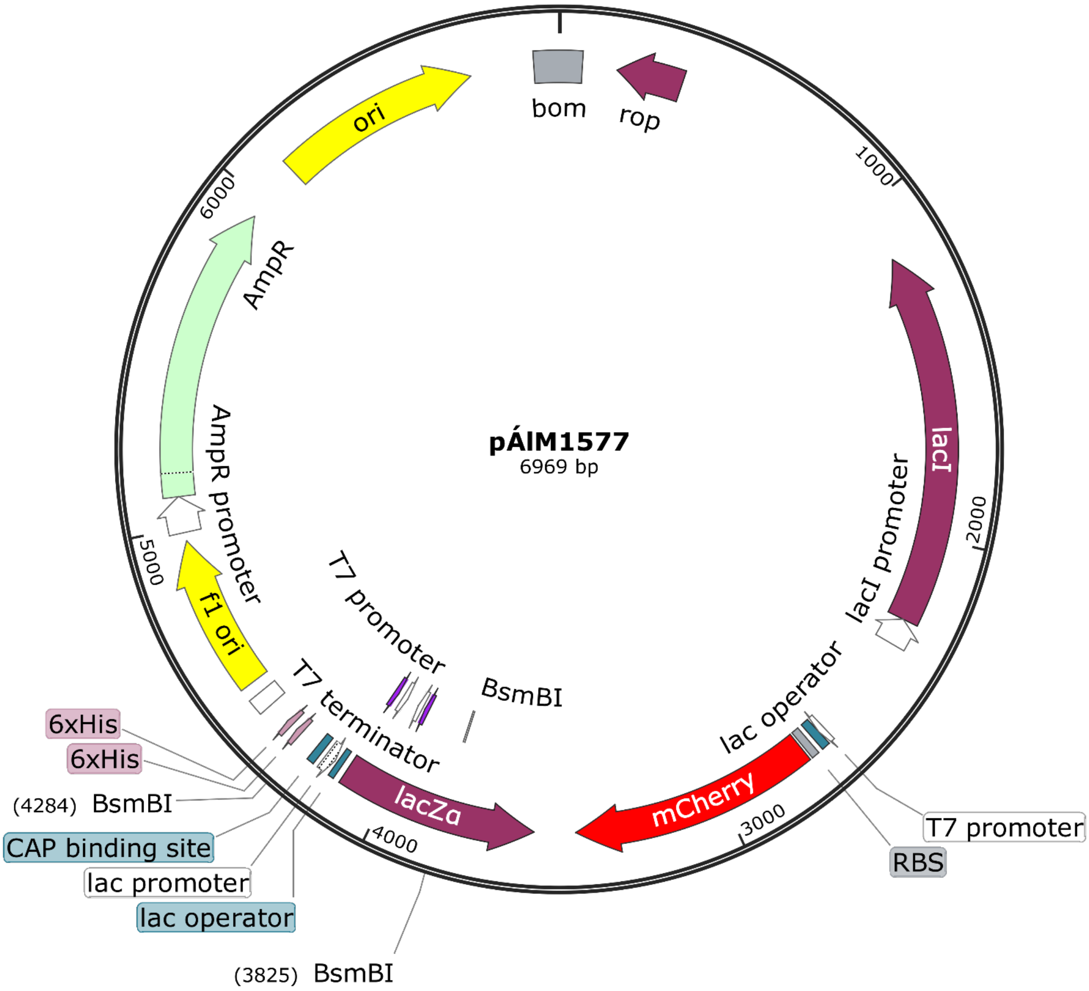

pÁlM1577

Vector for bacterial expression of TALEs fused to N-terminal mCherry and C-terminal 6xHis for protein purification.

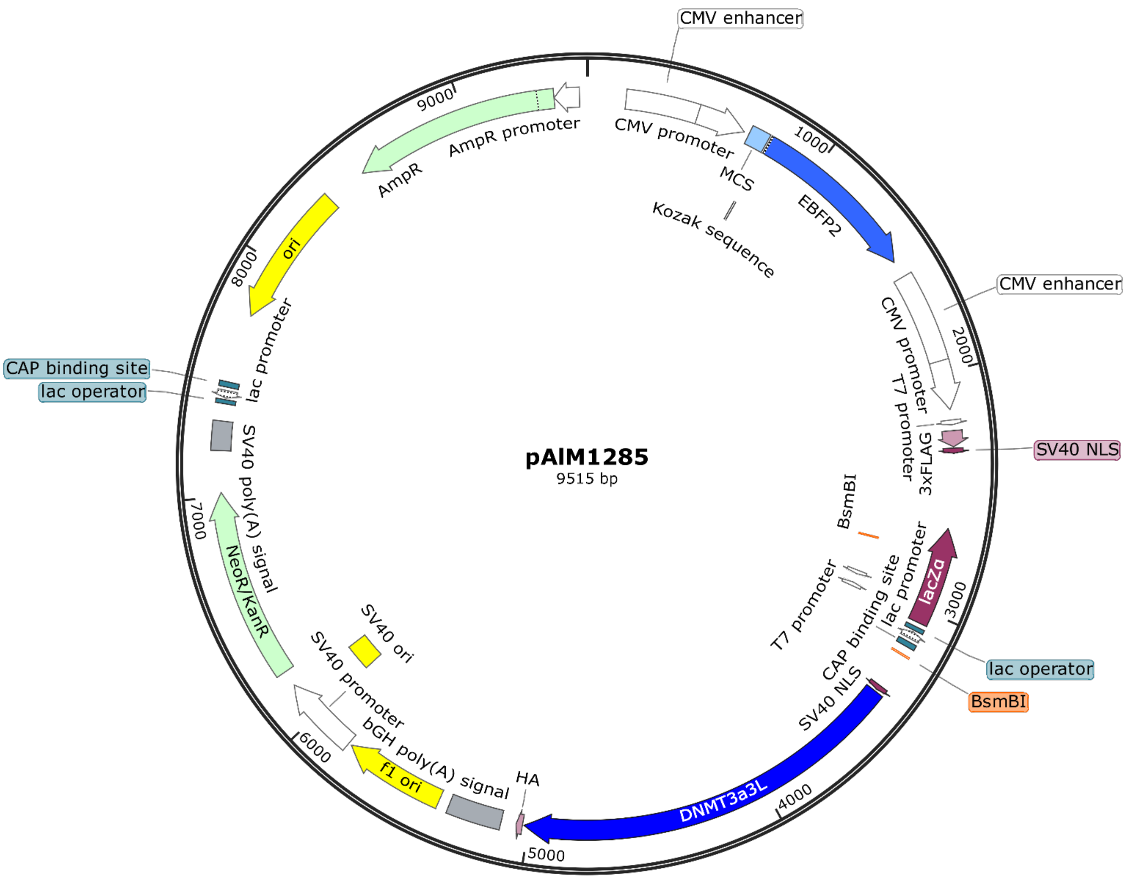

pÁlM1285

Vector for mammalian expression of TALEs fused to C-terminal DNMT3a3L and HA-tag and N-terminal 3XFLAG-tag for for live-cell site-specific DNA methylation. It also contains an EBFP2 fluorophore under the same promoter (CMV) as transfection control.

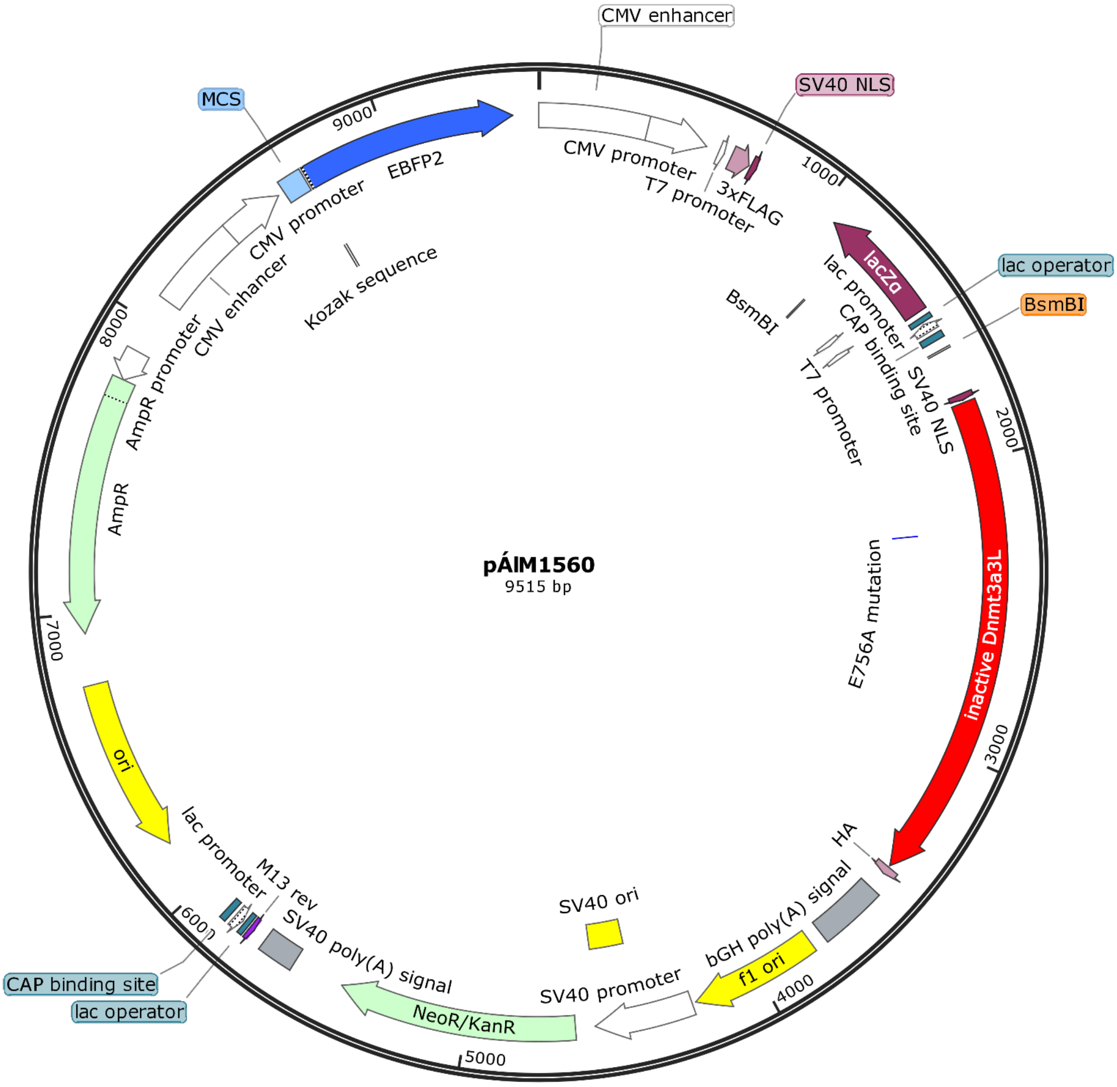

pÁlM1560

Vector for mammalian expression of TALEs fused to C-terminal inactive DNMT3a3L (carrying E756A mutation) and HA-tag and N-terminal 3XFLAG-tag. It also contains an EBFP2 fluorophore under the same promoter (CMV) as transfection control. This construct is used as negative control for site-specific DNA-methylation.

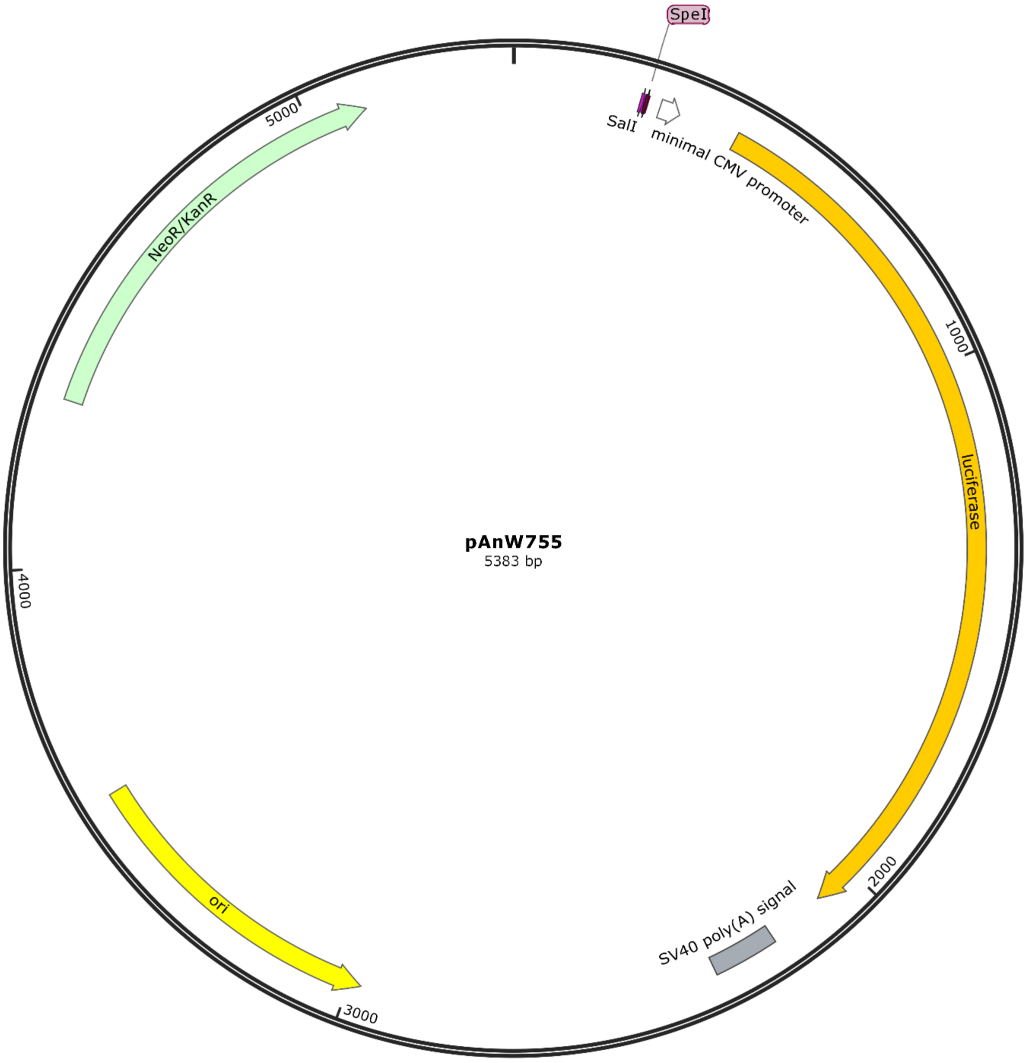

pAnW755

Plasmid map of luciferase reporter plasmid pAnW755. Shown are the restriction sites SalI (pink: SalI) and SpeI (purple: SpeI) for insertion of the TALE target sequence, a minimal CMV promoter (white: minimal CMV promoter) for luciferase expression (orange: luciferase) with a C-terminal SV40 poly(A) signal (grey: SV40 poly(A) signal), the origin for bacterial replication (yellow: ori) and the gene for neomycin/kanamycin resistance (bright green: NeoR/KanR).
